# A single oral glucose load decreases arterial plasma [K^+^] during exercise and recovery

**DOI:** 10.14814/phy2.14889

**Published:** 2021-06-10

**Authors:** Collene H. Steward, Robert Smith, Nigel K. Stepto, Malcolm Brown, Irene Ng, Michael J. McKenna

**Affiliations:** ^1^ Institute for Health and Sport Victoria University Melbourne VIC Australia; ^2^ Department of Anaesthesia Western Hospital Melbourne VIC Australia; ^3^ Department of Biochemistry and Pharmacology University of Melbourne Melbourne VIC Australia; ^4^ Department of Anaesthesia and Pain Management Royal Melbourne Hospital Melbourne VIC Australia

**Keywords:** exercise, fatigue, insulin, Na+,K+‐ATPase, oral glucose tolerance test, potassium

## Abstract

**Aim:**

We investigated whether acute carbohydrate ingestion reduced arterial potassium concentration ([K^+^]) during and after intense exercise and delayed fatigue.

**Methods:**

In a randomized, double‐blind crossover design, eight males ingested 300 ml water containing 75 g glucose (CHO) or placebo (CON); rested for 60 min, then performed high‐intensity intermittent cycling (HIIC) at 130% ${\dot{V}O}_{\text{2peak}}$, comprising three 45‐s exercise bouts (EB), then a fourth EB until fatigue. Radial arterial (a) and antecubital venous (v) blood was sampled at rest, before, during and after HIIC and analyzed for plasma ions and metabolites, with forearm arteriovenous differences (a‐v diff) calculated to assess inactive forearm muscle effects.

**Results:**

Glucose ingestion elevated [glucose]_a_ and [insulin]_a_ above CON (*p* = .001), being, respectively, ~2‐ and ~5‐fold higher during CHO at 60 min after ingestion (*p* = .001). Plasma [K^+^]_a_ rose during and declined following each exercise bout in HIIC (*p* = .001), falling below baseline at 5 min post‐exercise (*p* = .007). Both [K^+^]_a_ and [K^+^]_v_ were lower during CHO (*p* = .036, *p* = .001, respectively, treatment main effect). The [K^+^]_a‐v diff_ across the forearm widened during exercise (*p* = .001), returned to baseline during recovery, and was greater in CHO than CON during EB1, EB2 (*p* = .001) and EB3 (*p* = .005). Time to fatigue did not differ between trials.

**Conclusion:**

Acute oral glucose ingestion, as used in a glucose tolerance test, induced a small, systemic K^+^‐lowering effect before, during, and after HIIC, that was detectable in both arterial and venous plasma. This likely reflects insulin‐mediated, increased Na^+^,K^+^‐ATPase induced K^+^ uptake into non‐contracting muscles. However, glucose ingestion did not delay fatigue.

## Graphical abstract

Acute glucose intake substantially elevated insulin, with a systemic K^+^‐lowering effect evident in arterial and antecubital venous plasma before, during, and post‐exercise. An increased arterio‐venous K^+^ difference across the relatively inactive forearm musculature with glucose ingestion was consistent with increased K^+^ uptake in non‐active muscle; this was likely mediated via insulin‐induced activation of sodium‐potassium ATPase.

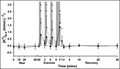

## 1 INTRODUCTION

Ongoing K^+^ regulation is vital to preserve excitability and contractile function in skeletal, as well as cardiac muscle (Lindinger & Cairns, 2021; Sejersted & Sjøgaard, 2000). In contracting skeletal muscle, the depolarization phase of action potentials is linked with cellular K^+^ efflux, resulting in increased interstitial [K^+^], decreased intracellular [K^+^] and membrane depolarization; these changes are proposed as one factor in muscular fatigue (Lindinger & Cairns, 2021; McKenna et al., 2008). Experiments utilizing in vitro isolated muscle preparations demonstrate that high extracellular [K^+^] strongly depresses maximal force (Cairns et al., 1997; de Paoli et al., 2007). It is fundamentally important therefore, that the Na^+^,K^+^‐ATPase (NKA), which plays a central role in acutely regulating K^+^ homeostasis in all tissues (Lindinger & Cairns, 2021), is also rapidly activated in contracting skeletal muscle, transporting K^+^ back into the intracellular compartment. Even brief muscle contractions activate the muscle NKA, which attenuates the excitation‐induced decline in intracellular [K^+^] and rise in intracellular [Na^+^], and exerts an electrogenic effect on the membrane potential (Clausen, 2008). Despite this, muscle contractions induce elevations in muscle interstitial [K^+^] (Green et al., 2000; Juel et al., 2000; Nordsborg et al., 2003) and in arterial plasma [K^+^], which can reach ~6–8 mM (Atanasovska et al., 2014, 2018; Clausen, 2008; Sejersted & Sjøgaard, 2000). Furthermore, upon cessation of exercise, plasma [K^+^] rapidly falls to rest or below resting concentrations within the first minutes of recovery, due to rapid re‐uptake of K^+^ by previously active muscle (Atanasovska et al., 2014; Lindinger & Cairns, 2021; Lindinger et al., 1992).

Insulin is also an important regulator of skeletal muscle NKA and thus also of K^+^ homeostasis. Early reports indicated that insulin lowered plasma [K^+^] in humans (Alvestrand et al., 1984; Andres et al., 1962; DeFronzo, 1988; DeFronzo et al., 1980; Ferrannini et al., 1988; Zierler & Rabinowitz, 1964), reflecting K^+^ uptake into skeletal muscle (Clausen, 1998; Clausen & Flatman, 1987; Ewart & Klip, 1995; Lindinger & Cairns, 2021). Insulin stimulates NKA in isolated muscles and membrane extracts (Clausen & Kohn, 1977; Erlij & Grinstein, 1976; Gavryck et al., 1975; McKenna et al., 2003; Moore, 1973), independently of increased muscle glucose transport (Clausen, 1986). In isolated rat soleus muscle, insulin stimulation for only 10 min resulted in a 3‐fold increase in NKA α‐subunit phosphorylation (Chibalin et al., 2001), via activation of protein kinase C (Chibalin et al., 2001; Sampson et al., 1994). In rat adipocytes, insulin stimulation of NKA has been attributed to an increased Na^+^ affinity of NKA α_1_‐ and α_2_‐isoforms (Ewart & Klip, 1995; McGill & Guidotti, 1991). The NKA cycle relies on ATP derived from glycogenolysis‐glycolysis (Dutka & Lamb, 2007; Jensen et al., 2020), with increased NKA activity linked with increased lactate (Lac^−^) production in skeletal muscle (James et al., 1996). Insulin increased NKA activity in skeletal muscle (Clausen & Kohn, 1977) and stimulated ouabain‐suppressible muscle lactate release, indicating this increased lactate was due to NKA activation (Novel‐Chate et al., 2001). Thus, muscle glucose metabolism and lactate production are linked to muscle NKA activation.

Little research has investigated the effects of a physiological, hyperinsulinemic state on plasma [K^+^], such as induced by a standard oral glucose tolerance test, where 75 g glucose is ingested. This test induced hypokalemia at rest in healthy individuals in one study (Natali et al., 1993), but not in another (Muto et al., 2005), although decreased venous serum [K^+^] was found in hemodialysis patients (Muto et al., 2005). We utilized the OGTT here rather than a different glucose ingestion protocol, to explore whether the standard clinical tool itself modulated K^+^ homeostasis in healthy participants. It is possible that more pronounced effects of insulin on [K^+^] will be evident during high intensity exercise, when circulating [K^+^] is considerably higher, due to the expected insulin activation of NKA in skeletal muscle, noting also the expected adrenergic stimulation of NKA in muscle (Cairns & Borrani, 2015). We therefore investigated whether glucose‐induced hyperinsulinemia would lower plasma [K^+^] and elevate [Lac^−^] during each of rest, high‐intensity intermittent exercise and recovery. We further investigated whether a K^+^‐lowering effect might reflect increased K^+^ uptake into non‐contracting muscle and whether such K^+^‐lowering might be linked with delay in fatigue. We hypothesized that acute oral glucose ingestion would lower systemic plasma [K^+^] at rest and during high‐intensity exercise, with an increased arteriovenous [K^+^] difference across non‐contracting skeletal muscle, as well as reduce [Lac^−^] and delay the onset of fatigue.

## 2 MATERIALS AND METHODS

### 2.1 Participants

Eight recreationally active individuals (6 males and 2 females; age 24.8 ± 4.9 years; height 175.0 ± 9.8 cm; body mass 74.1 ± 11.0 kg; mean ± SD) gave their written informed consent to participate in the study, which was approved by the Victoria University Human Research Ethics Committee.

### 2.2 Experimental design

Participants attended the laboratory on six occasions. The first visit involved initial screening and an incremental exercise test on a cycle ergometer to determine peak oxygen consumption (${\dot{V}O}_{\text{2peak}}$). During the second visit, participants were familiarized with the high‐intensity intermittent cycling protocol. Participants then completed two variability trials, followed by the final two visits, which comprised the experimental and placebo trials. During the experimental trial, participants ingested a carbohydrate solution (CHO) consisting of 75 g glucose, and in the placebo trial, an artificially sweetened placebo solution (CON) (NutraSweet) in 300 ml of water; both solutions were flavored with an unsweetened, caffeine‐free powder (Kool‐Aid, Kraft Foods). The CHO and CON trials were conducted in a double‐blind, randomized, crossover design. A one‐month delay between trials was to test female participants in the same phase of their menstrual cycle. The eight participants completed the two trials separated by 39 ± 12 d (mean ± SD), for the six males this was 41 ± 14 d (range 21–58 d) and for the two females was 32 and 34 d. Participants were asked to maintain current activity/exercise levels between trials and all participants verbally confirmed this, however formal activity logs were not recorded.

### 2.3 Incremental exercise testing

The ${\dot{V}O}_{\text{2peak}}$ was measured during an incremental test to volitional exhaustion conducted on an electronically braked cycle ergometer (Lode), at a cadence of 70 rpm. The ergometer was modified to have the participant in a partially recumbent position seated on a custom‐made chair, and was consistent for all subsequent trials. Participants sequentially completed four, 4‐min submaximal work periods at 60, 90, 120, and 150 W, followed by a 5‐min rest period. Exercise then recommenced at 175 W and was increased by 25 W every min until volitional exhaustion, defined as an inability to maintain a pedaling cadence above 60 rpm. Participants breathed through a Hans Rudolph two‐way non‐rebreathing valve, with expired air passing through low resistance tubing into a 4‐L mixing chamber. Expired airflow was measured using a flow transducer (K520, KL Engineering); fractions of expired O_2_ and carbon dioxide (CO_2_) were measured continuously by rapidly responding analyzers (Ametek S‐3A/II and Ametek CD‐3A, AEI Technologies). The ${\dot{V}O}_{2}$ was calculated continuously and displayed every 15 s on a personal computer (Turbofit, Vacumed). The ventilometer and gas analyzers were calibrated prior to each test with a standard 3‐L syringe and precision reference gases. The ${\dot{V}O}_{\text{2peak}}$ was calculated as the mean of the two highest consecutive 15 s values. A regression equation of ${\dot{V}O}_{2}$ versus power output was derived from the four submaximal workloads and the ${\dot{V}O}_{\text{2peak}}$ and used to determine a power output corresponding to 130% ${\dot{V}O}_{\text{2peak}}$ for each individual (Medved et al., 2003).

### 2.4 High‐intensity intermittent cycling

The high‐intensity intermittent cycling (HIIC) protocol comprised four exercise bouts (EB), each performed at a power output corresponding to 130% ${\dot{V}O}_{\text{2peak}}$ power output; the first three EBs (EB1–3) were each of 45 s duration, whilst the final EB (EB4) was continued until volitional exhaustion, defined as the inability to maintain a pedaling cadence above 50 rpm. All EB had an intervening 135‐s rest period, giving a 1:3 work‐to‐rest ratio. During the two variability sessions, the time to fatigue during the final bout (EB4) was measured to determine their individual performance variability.

### 2.5 Experimental trials

#### 2.5.1 Participant preparation

All participants arrived in the lab at 8 a.m. after an overnight fast and were allowed water *ad libitum*. Heart rate and rhythm were monitored via telemetry using a 12‐lead electrocardiogram (Mortara). A 20G arterial catheter (Mayo Healthcare) was inserted retrograde into the radial artery under local anaesthesia (1% Xylocaine, AstraZeneca), and a 20G catheter was inserted anterograde into the antecubital vein of the contralateral arm. Both catheters were connected to a saline filled arterial infusion kit (ITL Healthcare) and kept patent by a slow pressurized infusion of 0.9% sodium chloride.

#### 2.5.2 Blood sampling and analyses

Following cannulation participants rested for 30 min and the first blood sample was then taken (baseline). The participant then ingested either the CHO or CON solution and for the following 60 min remained passive in a supine position, with arterial and venous blood samples taken simultaneously at 10, 20, 40 and 60 min following ingestion. The participant was then moved to an adjacent cycle ergometer where arterial (a) and venous (v) samples were drawn simultaneously, immediately prior to and during the final seconds of EB1–4, and at 1, 2, 5, 10, 20 and 30 min during recovery. Contractions of the forearm musculature of the arm undergoing arterial and venous sampling were minimized throughout the trial by securing the supinated forearm and wrist to an inflexible brace, which rested on the cycle ergometer handlebars; this prevented wrist flexion and gripping of the handlebars and together with the semi‐recumbent position enabled the forearm musculature to be relatively inactive.

At each time point, two samples were taken from each site. The first sample (~0.6 ml) was drawn into a blood gas syringe (Seimens Rapidlyte lithium heparin) for immediate analysis of plasma K^+^, Na^+^ and pH using an automated blood gas analyzer (Ciba Corning 865, Seimens). The second sample (~3 ml) was drawn into a plain, latex‐free syringe and expelled into a tube containing lithium heparin (125 IU). Immediately, 400 µl of whole blood was transferred into a 1.5 ml microfuge tube for analysis of hemoglobin concentration ([Hb]) and hematocrit (Hct) using an automated analyzer (Sysmex Automated Cell Counter, Roche Diagnostics). The remaining blood was centrifuged at 4500 rpm for 2 min and the separated plasma was removed and stored at −20°C for later analyses of plasma glucose and Lac^−^ concentrations (YSI 2300 STAT plus Glucose Lactate Analyzer, John Morris Scientific). Plasma insulin concentration was measured in arterial blood samples taken at baseline, 20, 40, 60 min, and at fatigue, using an enzyme‐linked immunosorbent assay (ELISA) (DAKO).

##### Calculations

Changes were calculated from baseline in arterial and venous plasma volume (∆PV_a_, ∆PV_v_) and blood volume (∆BV_a_, ∆BV_v_), and in venous compared with arterial PV (∆PV_a‐v_) and BV (∆BV_a‐v_) across the forearm, from changes in [Hb] and Hct (Harrison, 1985; McKenna et al., 1997). Plasma hydrogen concentration (nmol.L^−1^) was calculated from measured pH. The changes in plasma [K^+^] from baseline (∆[K^+^] ∆[K^+^]) and across the forearm (∆[K^+^]_a‐v_) were calculated and corrected for fluid shifts using the corresponding calculated ∆PV, for each trial.

### 2.6 Statistical analyses

All results are expressed as mean ± SD. All data was tested for normality using the Shapiro‐Wilk *W* test. When normality criteria were not met (*p* < .05), data was log transformed to reduce bias of the error. Balanced data sets (no missing variables) were analyzed using a two‐way repeated measure ANOVA. Data sets that contained missing values were analyzed using a linear mixed model, with time and treatment (CHO or CON) as fixed effects, and restricted maximum likelihood as the estimation method for missing values. For each variable, two covariance methods were tested (first‐order autoregressive and compound symmetry), the appropriate structure was chosen by comparing the Aikaike Information Criterion (AIC) for each covariance type. Least Significant Difference post‐hoc tests were used for all analyses. Time‐by‐treatment interactions were not significant unless stated. Individual coefficients of variation were calculated for all subjects within the exercise protocol and averaged to obtain an overall coefficient of variation (CV) (Medved et al., 2003). Significance was accepted at *p* < .05. Statistical analyses were performed using PASW Statistics 20 (IBM SPSS Statistics).

## 3 RESULTS

### 3.1 Exercise performance

The participant's incremental exercise ${\dot{V}O}_{\text{2peak}}$ was 3.33 ± 0.52 L.min^−1^ (45.5 ± 8.6 ml.kg^−1^.min^−1^), peak power output was 293 ± 47 W, and the calculated work rate corresponding to 130% ${\dot{V}O}_{\text{2peak}}$ was 360 ± 75 W. Time to fatigue during EB4 was consistent in the two variability trials (70.0 ± 17.5 and 73.0 ± 21.9 s respectively), CV 6.6% (*p* = .54) and did not differ between experimental trials (CON 64.7 ± 19.7 vs. CHO 61.9 ± 17.7 s, *p* = .41).

### 3.2 Plasma [glucose]

#### 3.2.1 Arterial [glucose]

**FIGURE 1:**
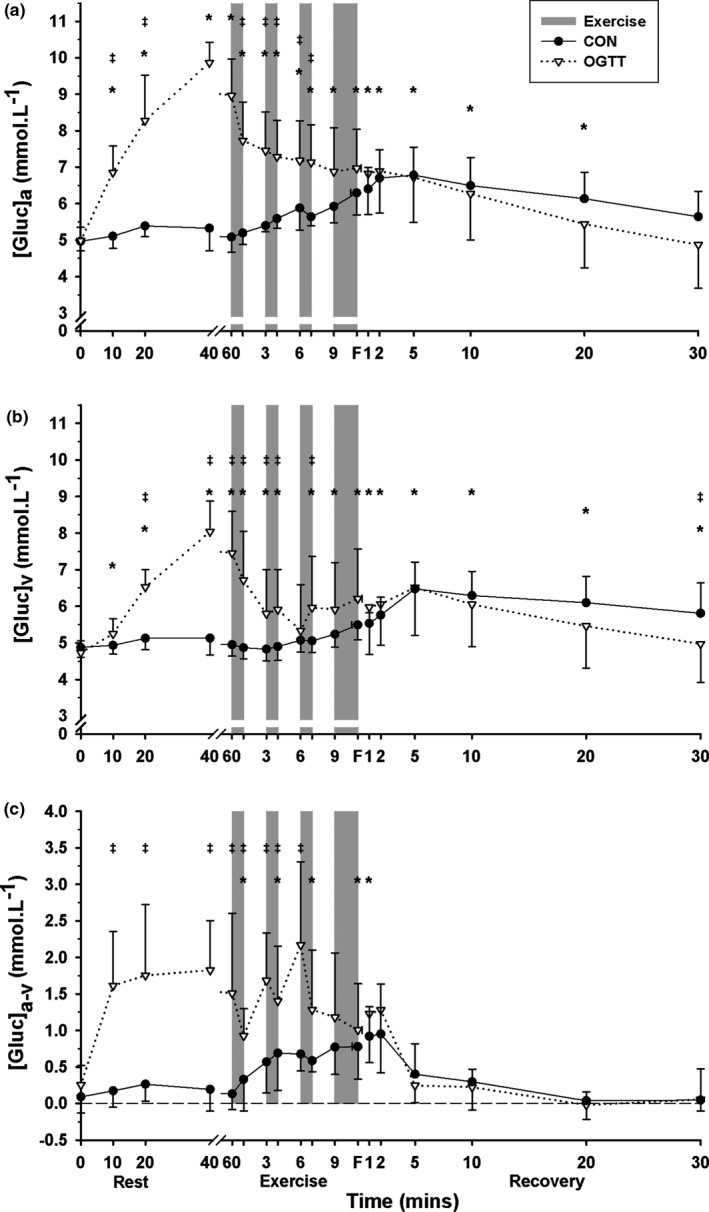
Effects of an oral glucose load on plasma [glucose] measured at baseline (0), and subsequent 60 min rest, during high intensity intermittent cycling at 130% ${\dot{V}O}_{\text{2peak}}$ continued to fatigue, and in 30 min recovery, following either carbohydrate (CHO), or placebo (CON) ingestion. (a) Arterial (a), (b) venous (v), and (c) calculated arterio‐venous differences (a‐v) in plasma [Gluc], under CON (●) and CHO (▽) conditions. *Different from baseline (*p* < .05, Time main effect). ^†^CHO different from CON (*p* < .05, Treatment main effect). ^‡^CHO greater than CON at that timepoint (*p* < .05). All data mean ± SD, *n* = 8. Shaded bars represent exercise bout (EB), with EB1–EB3 of 45 s duration each and EB4 continued until fatigue (F). Data were not corrected for fluid shifts

The [glucose]_a_ rose above baseline at 10 min rest and remained elevated, at 20 min post‐fatigue (*p* = .001, time main effect) and was greater during CHO than CON (*p* = .001, treatment main effect, Figure 1). The time‐by‐treatment interaction was significant (*p* = .001) and post‐hoc tests revealed [glucose]_a_ during CHO was greater than CON from 10 min after ingestion until end‐EB3, but did not differ thereafter (Figure 1).

#### 3.2.2 Venous [glucose]

A similar temporal pattern was observed for [glucose]_v_ (*p* = .001, time main effect), also being greater during CHO than CON (*p* = .006, treatment main effect, Figure 1). The time‐by‐treatment interaction was significant (*p* = .001), with [glucose]_v_ greater in CHO than CON from 20 min after ingestion through until EB2 and during EB3, before reversing to less than CON at 30 min recovery (Figure 1).

#### 3.2.3 Arteriovenous [glucose] difference

The [glucose]_a‐v_ was elevated above baseline indicating a large net glucose uptake into the forearm during each of EB1–EB4 and at 1 min recovery (*p* = .001, time main effect), with [glucose]_a‐v_ more positive during CHO than CON (*p* = .001, treatment main effect, Figure 1). The time‐by‐treatment interaction was significant (*p* = .001) with [glucose]_a‐v_ more positive in CHO than CON from 10 min post‐ingestion through to pre‐EB3, indicating a large net glucose uptake into the forearm at rest and during EB1–2 (Figure 1).

### 3.3 Arterial plasma insulin concentration

Arterial plasma [insulin]_a_ was increased above baseline at all time points (*p* = .001, time main effect) and was greater during CHO than CON (*p* = .001, treatment main effect, Table 1). There was a significant time‐by‐treatment interaction (*p* = .001); during CHO, [insulin]_a_ was elevated four‐ to fivefold above baseline at 20, 40 and 60 min (*p* = .005), remained more than twofold greater at fatigue (*p* = .01), and was greater than CON at 20, 40 and 60 min (*p* = .01) and at fatigue (*p* = .04, respectively, Table 1).

**TABLE 1:** Arterial plasma insulin concentration (pmol.L^−1^) measured at baseline, subsequent 60 min rest and at the point of fatigue during high intensity intermittent exercise at 130% ${\dot{V}O}_{\text{2peak}}$, following either carbohydrate (CHO), or placebo (CON) ingestion

	Baseline	Rest			Fatigue
		20 min	40 min	60 min	
CON	60.0 ± 29.6	62.9 ± 21.0	65.1 ± 52.6	42.6 ± 15.5	60.0 ± 17.5
CHO^a^	43.8 ± 10.2	209.0 ± 84.4^b^, ^c^	261.0 ± 85.6^b^, ^c^	212.7 ± 84.4^b^, ^c^	129.4 ± 33.5^b^, ^d^

Mean ± SD, *n* = 8.

^a^ Treatment main effect (*p* = .001).

^b^ CHO greater than baseline (*p* = .01).

^c^ CHO greater than CON at that time point (*p* = .005).

^d^ CHO greater than CON at that time point (*p* = .01).

### 3.4 Plasma [K^+^]

#### 3.4.1 Arterial [K^+^]

**FIGURE 2:**
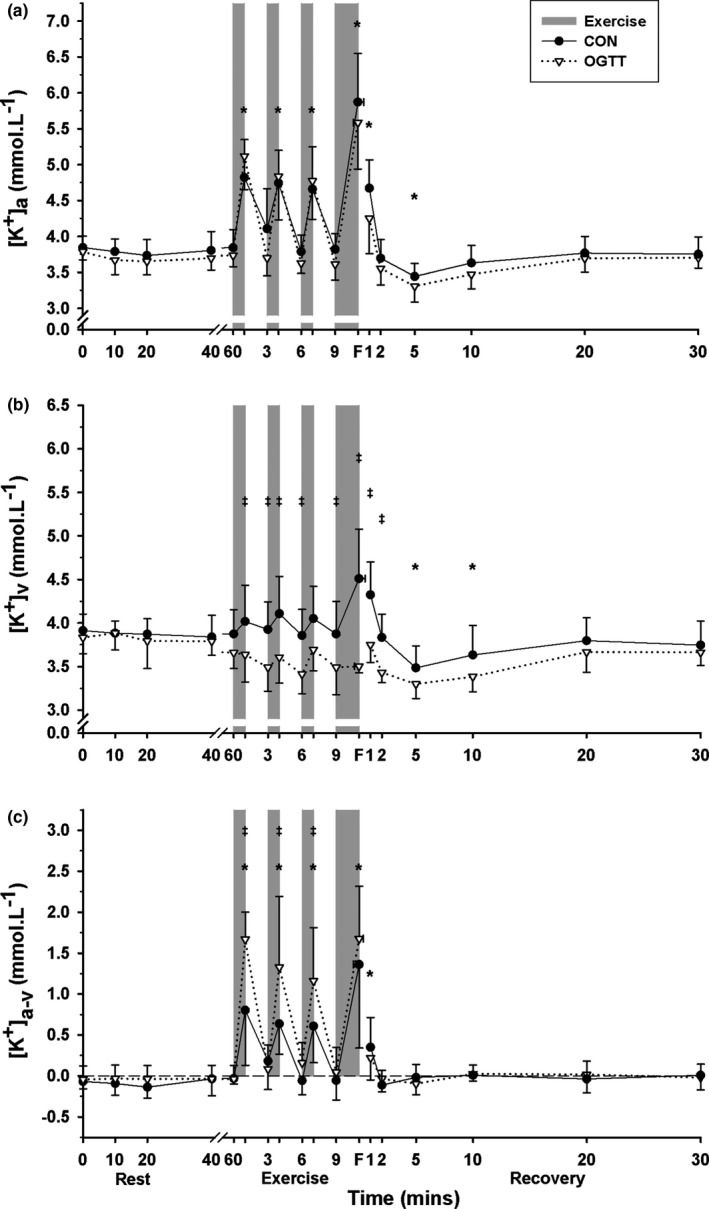
Effects of an oral glucose load on plasma [K^+^] measured at baseline (0), and subsequent 60 min rest, during high intensity intermittent cycling at 130% ${\dot{V}O}_{\text{2peak}}$ continued to fatigue, and in 30 min recovery, following either carbohydrate (CHO), or placebo (CON) ingestion. (a) Arterial (a), (b) venous (v), and (c) calculated arterio‐venous differences (a‐v) in plasma [K^+^], under CON (●) and CHO (▽) conditions. *Different from baseline (*p* < .05, Time main effect). ^†^CHO different from CON (*p* < .05, Treatment main effect). ^‡^CHO different from CON at that timepoint (*p* < .05). All data mean ± SD, *n* = 8. Shaded bars represent exercise bout (EB), with EB1–EB3 of 45 s duration each and EB4 continued until fatigue (F). Data were not corrected for fluid shifts

Plasma [K^+^]_a_ was elevated above baseline at the end of each EB, remained elevated at 1 min post‐fatigue, then fell below baseline at 5 min recovery (*p* = .001, time main effect); [K^+^]_a_ was lower in CHO than in CON (*p* = .036, treatment main effect, Figure 2).

#### 3.4.2 Venous [K^+^]

In contrast to [K^+^]_a_, plasma [K^+^]_v_ did not differ significantly from baseline during exercise, but did fall below baseline at 5 and 10 min post‐fatigue (*p* = .001, time main effect); [K^+^]_v_ was also lower in CHO than in CON (*p* = .001, treatment main effect, Figure 2). The time‐by‐treatment interaction was significant, with a lower [K^+^]_v_ during CHO than CON at EB1 through (except EB3) to 2 min post‐fatigue (*p* = .044, Figure 2).

#### 3.4.3 Arteriovenous [K^+^] difference

The [K^+^]_a‐v_ was greater (more positive) than baseline for each of EB1–4 and at 1 min post‐fatigue, representing a net K^+^ uptake into the relatively inactive forearm muscle (*p* = .001, time main effect); [K^+^]_a‐v_ was greater in CHO than CON (*p* = .001, treatment main effect, Figure 2). The time‐by‐treatment interaction was significant, with [K^+^]_a‐v_ more positive for CHO than CON during each of EB1–3 (*p* = .005), representing a greater net K^+^ uptake into the forearm during exercise in CHO, but with no differences in recovery (Figure 2).

### 3.5 Plasma [Na^+^]

#### 3.5.1 Arterial [Na^+^]

**FIGURE 3:**
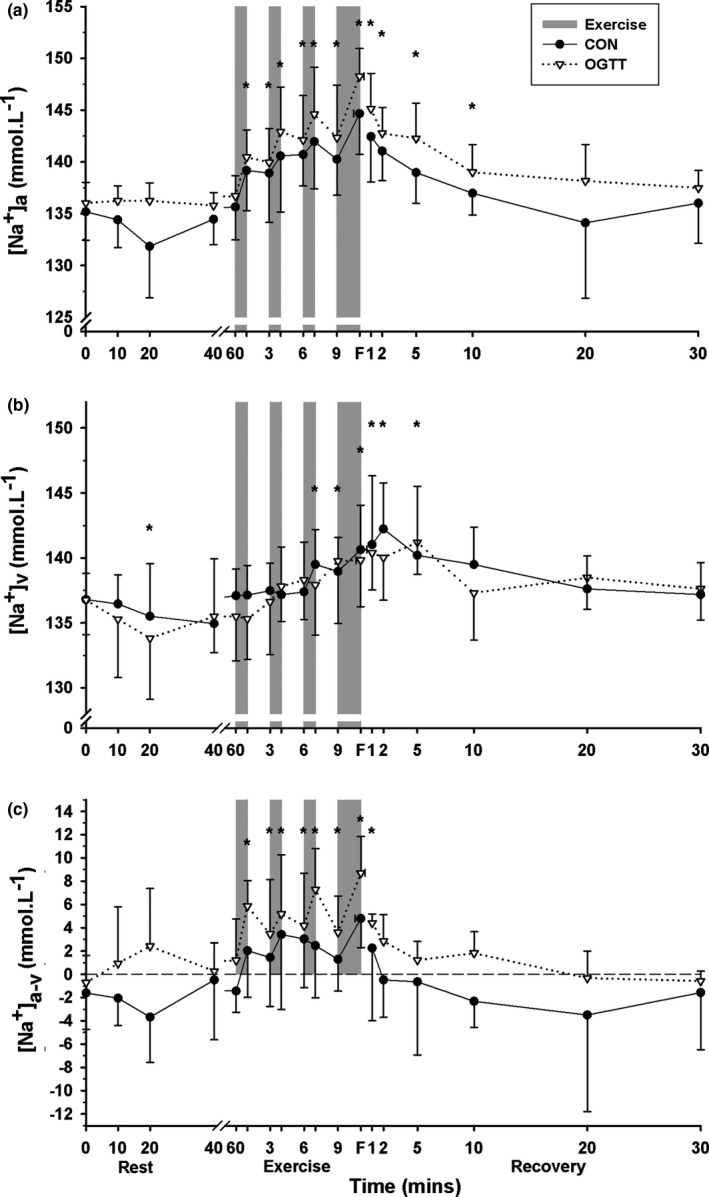
Effects of an oral glucose load on plasma [Na^+^] measured at baseline (0), and subsequent 60 min rest, during high intensity intermittent cycling at 130% ${\dot{V}O}_{\text{2peak}}$ continued to fatigue, and in 30 min recovery, following either carbohydrate (CHO), or placebo (CON) ingestion. (a) Arterial (a), (b) venous (v), and (c) calculated arterio‐venous differences (a‐v) in plasma [Na^+^], under CON (●) and CHO (▽) conditions. *Different from baseline (*p* < .05, Time main effect). ^†^CHO different from CON (*p* < .05, Treatment main effect). All data mean ± SD, *n* = 8. Shaded bars represent exercise bout (EB), with EB1–EB3 of 45 s duration each and EB4 continued until fatigue (F). Data were not corrected for fluid shifts

Plasma [Na^+^]_a_ was elevated above baseline from EB1 to 10 min recovery (*p* = .001, time main effect); [Na^+^]_a_ was greater during CHO than CON (*p* = .013, treatment main effect, Figure 3).

#### 3.5.2 Venous [Na^+^]

Venous [Na^+^] fell slightly at 20 min rest and was then elevated above baseline from EB3 through until 5 min post‐fatigue (*p* = .001, time main effect), with no differences between trials (*p* = .469, Figure 3).

#### 3.5.3 Arteriovenous [Na^+^] difference

The [Na^+^]_a‐v_ was more positive than baseline from EB1 to 1 min recovery (*p* = .001, time main effect). The [Na^+^]_a‐v_ was more positive in CHO than in CON representing a greater net Na^+^ uptake into the forearm (*p* = .001, treatment main effect, Figure 3).

### 3.6 Plasma [Lac^−^]

#### 3.6.1 Arterial [Lac^−^]

**FIGURE 4:**
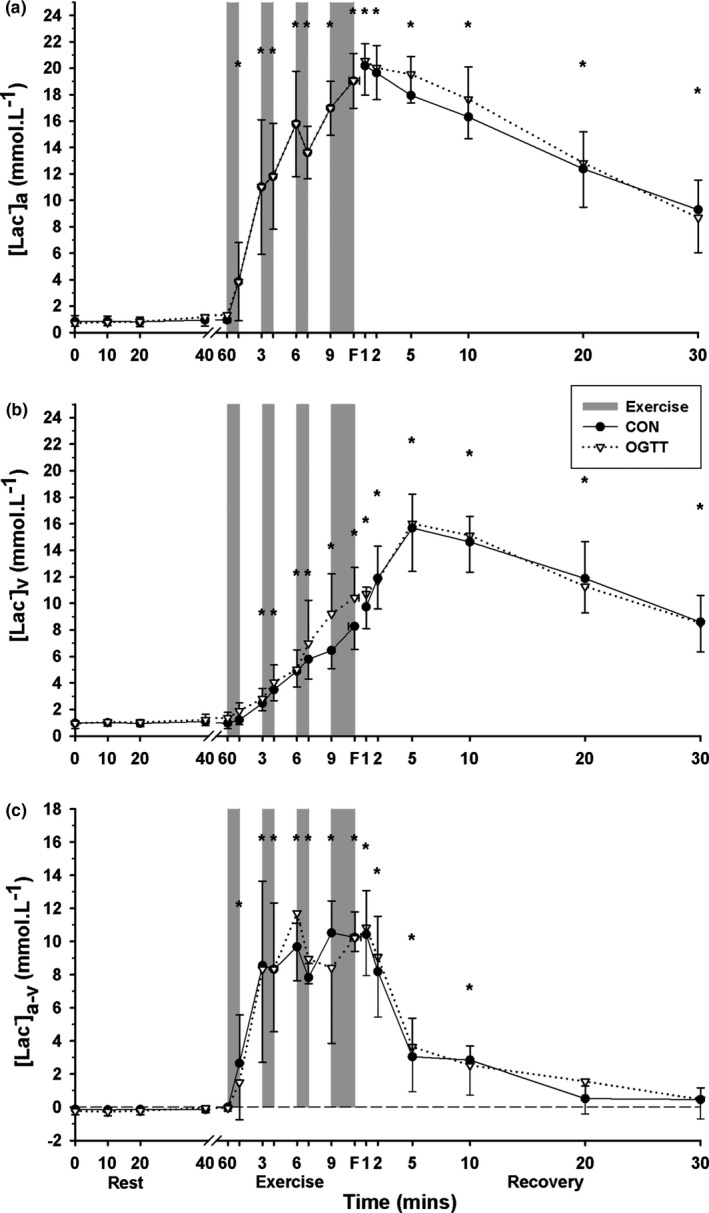
Effects of an oral glucose load on plasma [Lac^−^] measured at baseline (0), and subsequent 60 min rest, during high intensity intermittent cycling at 130% ${\dot{V}O}_{\text{2peak}}$ continued to fatigue, and in 30 min recovery, following either carbohydrate (CHO), or placebo (CON) ingestion. (a) Arterial (a), (b) venous (v), and (c) calculated arterio‐venous differences (a‐v) in plasma [Lac^−^], under CON (●) and CHO (▽) conditions. *Different from baseline (*p* < .05, Time main effect). All data mean ± SD, *n* = 8. Shaded bars represent exercise bout (EB), with EB1–EB3 of 45 s duration each and EB4 continued until fatigue (F). Data were not corrected for fluid shifts

Plasma [Lac^−^]_a_ was elevated above baseline at EB1 through to 30 min recovery (*p* = .001, time main effect, Figure 4), but did not differ between CHO and CON trials (*p* = .629).

#### 3.6.2 Venous [Lac^−^]

Venous [Lac^−^] was elevated above baseline from pre‐EB2 through to 30 min recovery (*p* = .001, time main effect), with no difference between trials (*p* = .339, Figure 4).

#### 3.6.3 Arteriovenous [Lac^−^] difference

The [Lac^−^]_a‐v_ was increased (positive) from EB1 through to 10 min recovery, representing a large net uptake of lactate into the forearm (*p* = .001, time main effect), with no differences between trials (*p* = .822, Figure 4).

### 3.7 Plasma [H^+^]

#### 3.7.1 Arterial [H^+^]

**FIGURE 5:**
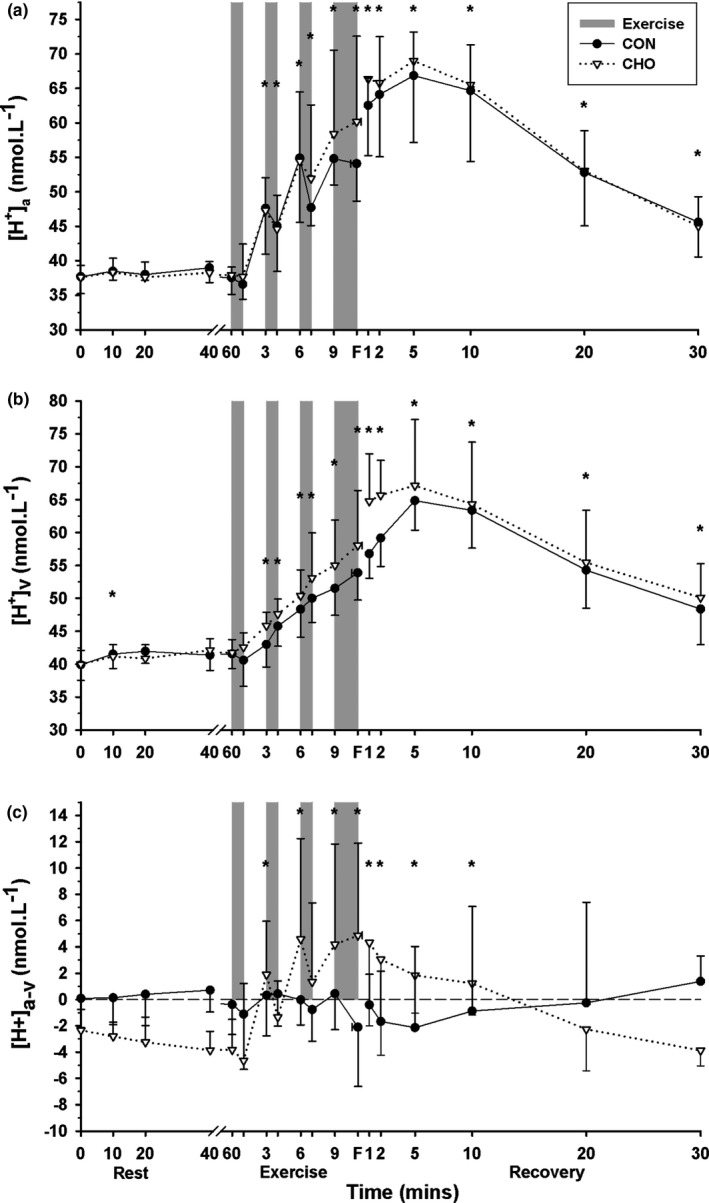
Effects of an oral glucose load on plasma [H^+^] measured at baseline (0), and subsequent 60 min rest, during high intensity intermittent cycling at 130% ${\dot{V}O}_{\text{2peak}}$ continued to fatigue, and in 30 min recovery, following either carbohydrate (CHO), or placebo (CON) ingestion. (a) Arterial (a), (b) venous (v), and (c) calculated arterio‐venous differences (a‐v) in plasma [H^+^], under CON (●) and CHO (▽) conditions. *Greater than baseline (*p* < .05; Time main effect). All data mean ± SD, *n* = 8. Shaded bars represent exercise bout (EB), with EB1–EB3 of 45 s duration each and EB4 continued until fatigue (F). Data were not corrected for fluid shifts

Plasma [H^+^]_a_ was elevated from pre‐EB2 until 30 min recovery (*p* = .001, time main effect) with no differences between CHO and CON trials (*p* = .739, Figure 5).

#### 3.7.2 Venous [H^+^]

The [H^+^]_v_ was increased at 10 min rest (*p* = .02), from pre‐EB2 until 30 min recovery (*p* = .001, time main effect), with no differences between CHO and CON (*p* = .345, Figure 5).

#### 3.7.3 Arteriovenous [H^+^] difference

The [H^+^]_a‐v_ was slightly greater (more positive) than baseline at pre‐EB2, pre‐EB3, pre‐EB4, EB4 and until 10 min post‐fatigue, (*p* = .001, time main effect), with no differences between CHO and CON (*p* = .458, Figure 5).

### 3.8 Hematology and fluid shifts

#### 3.8.1 Arterial and venous Hb and Hct

Arterial and venous [Hb] and Hct remained unchanged from baseline during 60 min rest, with exception of Hct_a_ which fell slightly at 20 min (*p* = .001); all variables then increased above baseline from EB1 through to 20 min recovery (*p* = .0001, time main effect, data not shown). There were no differences in [Hb] or Hct between CHO and CON (data not shown).

#### 3.8.2 Plasma volume changes

The changes in arterial plasma volume (∆PV_a_) comprised a slight increase at 20 min rest (*p* = .049), then decrease during exercise (*p* = .0001) by ~20% at EB4, before being gradually restored during recovery (*p* = .0001; time main effect, data not shown). There were no differences in ∆PV_a_ between CHO and CON (data not shown). The changes in venous plasma volume (∆PV_v_) comprised declines during exercise (*p* = .0001) and early recovery (*p* = .0001; time main effect), with no differences between trials (data not shown). The arteriovenous changes in plasma volume across the forearm (∆PV_a‐v_) were positive, indicating a small net gain in plasma volume across the forearm (*p* = .04; time main effect), but with no differences between trials (data not shown).

#### 3.8.3 Blood volume changes

Similar time main effects (*p* < .05) were found for ∆BV_a_, ∆BV_v_ or ∆BV_a‐v_ as reported for ∆PV, and there were no treatment or interaction effects (data not shown).

#### 3.8.4 Correction of [K^+^] for fluid shifts

The changes in plasma [K^+^] from baseline (∆[K^+^]_a_, ∆[K^+^]_v_) and across the forearm (∆[K^+^]_a‐v_) corrected for fluid shifts using the calculated ∆PV had very similar time and treatment main effects and interaction effects, as the non‐corrected plasma [K^+^]_a_, [K^+^]_v_ and [K^+^]_a‐v_ (data not shown).

## 4 DISCUSSION

The key finding of this study was that an acute oral glucose load routinely undertaken clinically via an oral glucose tolerance test and therefore under physiological conditions, affected systemic, and skeletal muscle K^+^ homeostasis during high‐intensity intermittent cycling (HIIC) exercise and recovery. Glucose ingestion lowered both arterial and antecubital venous plasma [K^+^] during rest, during and following HIIC (treatment main effect). In contrast to the small reductions in arterial plasma [K^+^] with CON, large reductions in [K^+^]_v_ were evident after CHO compared to placebo ingestion, during and immediately following HIIC. The [K^+^]_a‐v diff_ across the forearm was increased across all times after CHO ingestion, with the large positive increases during the first three exercise EB augmented with CHO (e.g., from ~0 mM at rest to ~0.8 mM during EB1 for CON and ~1.8 mM for CHO). This indicates greater net K^+^ uptake into the forearm musculature after CHO ingestion, assuming similar forearm blood flow in both trials. Thus, even a modest glucose load perturbs systemic [K^+^], with these effects amplified under conditions of severe physiological stress when [K^+^] is markedly elevated, such as intense leg cycling exercise. The greater [K^+^]‐lowering effects during exercise after glucose ingestion compared to placebo can most likely be attributed to the consequent physiological hyper‐insulinemia, via actions on NKA, including in inactive skeletal muscle. The marked [K^+^] lowering also evident after each exercise bout are also consistent with increased activity of muscle NKA.

### 4.1 Glucose ingestion and K^+^ homeostasis

Glucose ingestion doubled resting [glucose]_a_ to ~10 mmol.L^−1^, with the wide positive [glucose]_a‐v diff_ indicating a large net glucose uptake into the forearm, consistent with peripheral glucose disposal (McConell et al., 1994). The four‐ to fivefold increase in [insulin]_a_ above rest was sufficient to lower arterial [K^+^] across all times (treatment main effect), although the effect was small, such that no significant differences were observed between trials at any specific time point. Larger reductions were evident in [K^+^]_v_ with glucose ingestion, being markedly lower during the exercise period, with the exception of EB3, and in the first two minutes of recovery. Interestingly, the [K^+^]_v_ during the exercise period were close to, or even below concentrations classified as being hypokalemic (3.5 mmol.L^−1^) and were clearly so at 5–10 min recovery. Consequently, the [K^+^]_a‐v_ difference across the forearm muscles during exercise was increased by as much as 1.0 mmol.L^−1^ with CHO. These findings demonstrate that an oral glucose load elevating insulin to physiological concentrations also markedly affects K^+^ homeostasis during HIIC and early recovery. These findings extend previous reports that insulin infusion can elicit a decrease in plasma [K^+^] when measured under resting conditions (Alvestrand et al., 1984; Andres et al., 1962; DeFronzo et al., 1980). However, an important additional finding is that the effects of endogenous hyperinsulinemia on plasma [K^+^] were very small during the 60‐min rest post‐ingestion, such that we could not detect significant differences between the CHO and placebo conditions during this period. This means the minor [K^+^]‐lowering glucose‐insulin effects at rest were greatly amplified under conditions of intense intermittent exercise and in early recovery, when [K^+^] fluctuated markedly between baseline, well above baseline and hypokalemia in recovery. Similar findings occurred for ∆[K^+^] (data not reported).

This finding extends earlier studies that investigated the effects of beverages containing glucose on exercise performance and on measured [K^+^] in forearm venous blood in endurance athletes. Murray et al. found no effect of a 5% glucose polymer, or of mixed beverages containing either sucrose/glucose/electrolytes or fructose/glucose/electrolytes on venous [K^+^] during repeated submaximal cycling bouts at 55%–65% VO_2max_ and two subsequent performance tests at 90% and 75% VO_2max_ (Murray et al., 1987). Tarnopolsky et al. also found no effect of 8% glucose on venous [K^+^] during a one hour run at 75% VO_2max_ (Tarnopolsky et al., 1996). However, they did find lower venous [K^+^] during exercise after ingesting a mixed beverage pre‐exercise of 8% glucose polymer + fructose + electrolytes (5.7 mM [K^+^], 17.8 mM [Na^+^]) and then another beverage during exercise of 8% glucose polymer and fructose + glucose + electrolytes (0.8 mM [K^+^], 17.8 mM [Na^+^]) (Tarnopolsky et al., 1996). Given the large differences reported here between arterial and forearm venous [K^+^], the venous [K^+^] reported in both these studies (Murray et al., 1987; Tarnopolsky et al., 1996) would have been heavily influenced by the effects of the forearm musculature, which can substantially elevate [K^+^] if their activity is not controlled (Sostaric et al., 2006); in addition, forearm venous [K^+^] would also be expected to be influenced by adrenergic activity (Altarawneh et al., 2016).

An example of the physiological benefits of insulin stimulation of K^+^ uptake are demonstrated through consequential (extrarenal) effects of a high K^+^ ingestion with a meal, where the accompanying rise in glucose stimulates pancreatic insulin release which then via a feed forward mechanism induces cellular uptake of K^+^ in skeletal muscle (McDonough & Youn, 2017). This muscle K^+^ uptake acts to restrict the rise in plasma [K^+^] to prevent potentially life‐threatening effects of hyperkalemia on the myocardium, but also to conserve muscle intracellular K^+^ stores, which are essential K^+^ donors during periods of fasting, to preserve plasma [K^+^] and prevent deleterious effects of hypokalemia (McDonough & Youn, 2017). Post‐exercise hypokalemia has been identified as increasing the risk of arrhythmias (Lindinger & Cairns, 2021) and was recently shown to be correlated with QT hysteresis, indicating impaired cardiac repolarization and increased risk of arrhythmias (Atanasovska et al., 2018).

Thus, while plasma [K^+^] is regulated between renal excretion and extrarenal redistribution, skeletal muscle plays a vital role in the acute regulation of plasma [K^+^], especially during periods of elevated [K^+^], such as dietary K^+^ intake and exercise (McDonough & Youn, 2017). During the first hour of an insulin clamp conducted under resting conditions, around 30% of K^+^ uptake was attributable to peripheral tissues including skeletal muscles, with the splanchnic bed responsible for the remaining 70% (Andres et al., 1962). While the contracting musculature is responsible for the large rise in circulating [K^+^] during exercise (Lindinger & Cairns, 2021; Sejersted & Sjøgaard, 2000), inactive muscle also plays a key modulatory role on [K^+^]; the relatively inactive forearm muscles extract circulating K^+^ during leg cycling exercise, (Kowalchuk et al., 1988; Lindinger et al., 1990) thus attenuating the rise in plasma [K^+^] (Lindinger, 1995). The wide [K^+^]_a‐v_ across the forearm found here during HIIC is consistent with this role for the relatively inactive forearm muscles during leg cycling exercise. Importantly, the positive forearm [K^+^]_a‐v_ during HIIC was greatly amplified after glucose ingestion, suggesting that the glycemic effect on K^+^ homeostasis with intense exercise is also mediated largely by inactive skeletal muscle.

One limitation in our interpretations is that we did not measure forearm blood flow, which would have enabled determination of K^+^ uptake by the forearm during rest and HIIC. During a prolonged hyper‐insulinemic clamp under resting conditions, up to 50% increases in forearm blood flow and a ~doubling of the [glucose]_a‐v diff_ were reported (Fugmann et al., 1998). The small forearm [K^+^]_a‐v_ observed during pre‐exercise resting conditions for both CON and CHO do not suggest a strong effect on K^+^ uptake. Considerable variability has been reported for blood flow to inactive arm muscles during leg exercise. Early reports suggested initial biphasic increases in forearm blood flow were then normalized during low intensity exercise, but then declined during heavy leg exercise (Bevegard & Shepherd, 1966). More recent evidence points to intensity‐dependent increases in forearm blood flow during moderate to heavy intensity leg exercise (Green et al., 2002; Tanaka et al., 2006), with arm blood flow greatly increased at 2 min after supramaximal intensity cycling exercise (Medbø et al., 2009). Increases in flow have been suggested to aid the correction of arterial ionic disturbances with intense exercise (Lindinger et al., 1990). The lack of difference between CHO and CON treatments on [Hb] and Hct across the forearm suggests that glucose ingestion and the consequent insulinemia had no major effect on forearm blood flow during and after HIIC. However, with any increase in forearm blood flow during HIIC, K^+^ uptake would have been at least doubled after glucose ingestion, as in indicated by the greater (more positive) forearm [K^+^]_a‐v_. Thus, regardless of extent of increase in forearm blood flow during leg cycling exercise, the impacts of glycemia remain. Furthermore, the plasma volume shifts as blood traversed the forearm indicated a net gain in plasma water, indicating that the greater net K^+^ uptake across the forearm was not simply due to a hemoconcentration effect.

### 4.2 Glucose ingestion, lactate and sodium homeostasis, and performance

These glycemic‐induced effects on K^+^ were most likely due to insulin‐induced increases in NKA activity in the relatively inactive forearm musculature, as previously demonstrated in isolated skeletal muscle (Clausen & Kohn, 1977) and in the plasma membranes of rat skeletal muscles (Hundal et al., 1992; Marette et al., 1993). The NKA relies on glycolytically derived ATP supply (Dutka & Lamb, 2007; Jensen et al., 2020), increased NKA activity during sepsis has been linked with increased lactate production in skeletal muscle (James et al., 1996) and high plasma [glucose] has also been suggested to directly fuel glycolytic ATP for NKA (Dufer et al., 2009; Okamoto et al., 2001). We therefore determined whether increased endogenous insulin with glucose ingestion might also further elevate plasma [lactate] due to increased skeletal muscle NKA activity. However, we found no effects of glucose on plasma [Lac^−^] at rest, during or after HIIC, which suggests the contributions of NKA in inactive muscle on lactate concentration were minor. Since lactate is both produced and utilized by skeletal muscle fibers (Stainsby et al., 1991), it is possible that increased insulin‐induced lactate production with NKA activity could be countered by a simultaneous increase in lactate utilization (Novel‐Chate et al., 2001) within the same and/or surrounding cells. However, it is likely that any glycolytic effects of NKA stimulation were swamped by the increased metabolic lactate production in the contracting muscle (Hargreaves et al., 1998), consistent with a high peak plasma [lactate]_a_ of ~20 mmol.L^−1^. This similarly explains the lack of glucose effect on systemic [H^+^]. Nonetheless, our findings of a very large positive [Lac^−^]_a‐v_ difference across the forearm muscles during and after HIIC are consistent with inactive muscle playing an important role in [Lac^−^] regulation during intense exercise (Kowalchuk et al., 1988; Lindinger et al., 1990; Poortmans et al., 1978).

The increased [Na^+^]_a_ and positive [Na^+^]_a‐v diff_ across the forearm during leg cycling exercise are consistent with previous reports (Lindinger et al., 1990; McKenna, 1992). The small rise in [Na^+^]_a_ evident with exercise was proportionally less than the ∆PV, indicating an overall loss of Na^+^ from the circulation into extracellular and intracellular spaces in the contracting muscles (McKenna, 1992). The positive [Na^+^]_a‐v diff_ with exercise also indicates Na^+^ entry into non‐contracting muscle, consistent with an earlier finding of increased Na^+^ content in deltoid muscle during leg cycling exercise, although they did not detect significant increases in intracellular [Na^+^] (Lindinger et al., 1990). The greater [Na^+^]_a‐v diff_ across the forearm after glucose ingestion is most likely due to the higher [Na^+^]_a_, which would increase the plasma‐intracellular Na^+^ concentration gradient and drive Na^+^ into the forearm extracellular and intracellular spaces. Increased NKA activation in forearm muscles would not be expected to markedly affect plasma [Na^+^] due to the high extracellular [Na^+^] in both muscle and blood (McKenna, 1992). However, increased muscle NKA activity after glucose ingestion might be linked to increased Na^+^ uptake into inactive muscle in vivo, by facilitating Na^+^/glucose co‐transport into muscle, along with other Na^+^ transport mechanisms (Gagnon & Delpire, 2021).

Finally, the small effects of glucose ingestion on [K^+^]_a_ were not associated with any improvement in cycling time to fatigue during HIIC, suggesting insulin‐stimulated muscle NKA effects were insufficient to significantly enhance muscle function. Whilst previous research has demonstrated that oral glucose ingestion can enhance glucose utilization, improve exercise performance and attenuate fatigue, in humans (Coyle et al., 1983, 1986; Green et al., 2007) and animal models (Karelis et al., 2002), these studies typically examined prolonged submaximal exercise, where the attenuation of muscle fatigue is more closely aligned with increased [glucose] than increased plasma [insulin] (Karelis et al., 2003). This contrasts the HIIC protocol here, where factors such as glucose delivery to the contracting muscles are less likely to precipitate fatigue than impairments in sarcolemmal or t‐tubular excitability, or other sequelae from severe metabolic disturbances such as on sarcoplasmic reticulum Ca^2+^ release or the myofilaments.

## 5 CONCLUSIONS

Utilization of the standard oral glucose tolerance test protocol combined with high intensity intermittent exercise enabled examination of the effects of a physiological (endogenous) increase in plasma [insulin] on K^+^ and Lac^−^ regulation under rest, intense intermittent exercise and recovery conditions. Glucose ingestion lowered plasma [K^+^] across all times in both arterial and antecubital venous [K^+^], and during exercise resulted in a lower venous [K^+^] and widening of the arteriovenous [K^+^] difference, which likely reflect increased NKA activity in the inactive forearm musculature. Thus under physiological conditions, hyperglycemia can modulate systemic K^+^ homeostasis under resting and intense intermittent exercise conditions.

## CONFLICT OF INTEREST

No conflicts of interests are declared.

## AUTHOR CONTRIBUTIONS

Collene H. Steward—study design and preparation, data collection, statistical analysis, manuscript preparation. Robert Smith—data collection, manuscript preparation. Nigel K. Stepto—data collection, statistical analysis, manuscript preparation. Malcolm Brown—study design an preparation, data collection, manuscript preparation. Irene Ng—data collection, manuscript preparation. Michael J. McKenna—study design and preparation, data collection, statistical analysis, manuscript preparation.

## References

[phy214889-bib-0001] Altarawneh, M. , Petersen, A. C. , Smith, R. , Rouffet, D. M. , Billaut, F. , Perry, B. D. , Wyckelsma, V. L. , Tobin, A. , & McKenna, M. J. (2016). Salbutamol effects on systemic potassium dynamics during and following intense continuous and intermittent exercise. European Journal of Applied Physiology, 116, 2389–2399. 10.1007/s00421-016-3481-0 27771799

[phy214889-bib-0002] Alvestrand, A. , Wahren, J. , Smith, D. , & DeFronzo, R. A. (1984). Insulin‐mediated potassium uptake is normal in uremic and healthy subjects. American Journal of Physiology: Endocrinology and Metabolism, 246, E174–E180. 10.1152/ajpendo.1984.246.2.E174 6364842

[phy214889-bib-0003] Andres, R. , Baltzan, M. A. , Cader, G. , & Zierler, K. L. (1962). Effect of insulin on carbohydrate metabolism and on potassium in the forearm of man. Journal of Clinical Investigation, 41, 108–115. 10.1172/JCI104452 PMC289199 13861460

[phy214889-bib-0004] Atanasovska, T. , Petersen, A. C. , Rouffet, D. M. , Billaut, F. , Ng, I. , & McKenna, M. J. (2014). Plasma K^+^ dynamics and implications during and following intense rowing exercise. Journal of Applied Physiology, 117, 60–68. 24812644 10.1152/japplphysiol.01027.2013

[phy214889-bib-0005] Atanasovska, T. , Smith, R. , Graff, C. , Tran, C. T. , Melgaard, J. , Kanters, J. K. , Petersen, A. C. , Tobin, A. , Kjeldsen, K. P. , & McKenna, M. J. (2018). Protection against severe hypokalemia but impaired cardiac repolarization after intense rowing exercise in healthy humans receiving salbutamol. Journal of Applied Physiology, 125, 624–633. 10.1152/japplphysiol.00680.2017 29745804

[phy214889-bib-0006] Bevegard, B. S. , & Shepherd, J. T. (1966). Reaction in man of resistance and capacity vessels in forearm and hand to leg exercise. Journal of Applied Physiology, 21, 123–132. 10.1152/jappl.1966.21.1.123 5903898

[phy214889-bib-0007] Cairns, S. P. , & Borrani, F. (2015). β‐Adrenergic modulation of skeletal muscle contraction: Key role of excitation–contraction coupling. Journal of Physiology, 593, 4713–4727. 10.1113/JP270909 PMC4626548 26400207

[phy214889-bib-0008] Cairns, S. P. , Hing, W. A. , Slack, J. R. , Mills, R. G. , & Loiselle, D. S. (1997). Different effects of raised [K+]o on membrane potential and contraction in mouse fast‐ and slow‐twitch muscle. American Journal of Physiology: Cell Physiology, 273, C598–C611. 10.1152/ajpcell.1997.273.2.C598 9277357

[phy214889-bib-0009] Chibalin, A. V. , Kovalenko, M. V. , Ryder, J. W. , Feraille, E. , Wallberg‐Henriksson, H. , & Zierath, J. R. (2001). Insulin‐ and glucose‐induced phosphorylation of the Na(+),K(+)‐adenosine triphosphatase alpha‐subunits in rat skeletal muscle. Endocrinology, 142, 3474–3482. 11459793 10.1210/endo.142.8.8294

[phy214889-bib-0010] Clausen, T. (1986). Regulation of active Na^+^‐K^+^ transport in skeletal muscle. Physiological Reviews, 66, 542–580. 10.1152/physrev.1986.66.3.542 3016768

[phy214889-bib-0011] Clausen, T. (1998). Clinical and therapeutic significance of the Na^+^,K^+^ pump. Clinical Science, 95, 3–17. 10.1042/cs0950003 9662481

[phy214889-bib-0012] Clausen, T. (2008). Role of Na^+^,K^+^‐pumps and transmembrane Na^+^,K^+^‐distribution in muscle function. The FEPS lecture ‐ Bratislava 2007. Acta Physiologica Scandinavica, 192, 339–349. 10.1111/j.1748-1716.2007.01798.x 17988242

[phy214889-bib-0013] Clausen, T. , & Flatman, J. A. (1987). Effects of insulin and epinephrine on Na^+^‐K^+^ and glucose transport in soleus muscle. American Journal of Physiology‐Endocrinology and Metabolism, 252, E492–E499. 10.1152/ajpendo.1987.252.4.E492 3031991

[phy214889-bib-0014] Clausen, T. , & Kohn, P. G. (1977). The effect of insulin on the transport of sodium and potassium in rat soleus muscle. Journal of Physiology, 265, 19–42. 10.1113/jphysiol.1977.sp011703 PMC1307806 850160

[phy214889-bib-0015] Coyle, E. F. , Coggan, A. R. , Hemmert, M. K. , & Ivy, J. L. (1986). Muscle glycogen utilization during prolonged strenuous exercise when fed carbohydrate. Journal of Applied Physiology, 61, 165–172. 10.1152/jappl.1986.61.1.165 3525502

[phy214889-bib-0016] Coyle, E. F. , Hagberg, J. M. , Hurley, B. F. , Martin, W. H. , Ehsani, A. A. , & Holloszy, J. O. (1983). Carbohydrate feeding during prolonged strenuous exercise can delay fatigue. Journal of Applied Physiology, 55, 230–235. 10.1152/jappl.1983.55.1.230 6350247

[phy214889-bib-0017] de Paoli, F. V. , Overgaard, K. , Pedersen, T. H. , & Nielsen, O. B. (2007). Additive protective effects of the addition of lactic acid and adrenaline on excitability and force in isolated rat skeletal muscle depressed by elevated extracellular K^+^ . Journal of Physiology, 581, 829–839. 10.1113/jphysiol.2007.129049 PMC2075200 17347268

[phy214889-bib-0018] DeFronzo, R. A. (1988). Obesity is associated with impaired insulin‐mediated potassium uptake. Metabolism: Clinical and Experimental, 37, 105–108. 10.1016/S0026-0495(98)90001-4 3277011

[phy214889-bib-0019] DeFronzo, R. A. , Felig, P. , Ferrannini, E. , & Wahren, J. (1980). Effect of graded doses of insulin on splanchnic and peripheral potassium metabolism in man. American Journal Physiology: Endocrinology and Metabolism, 238, E421–E427. 10.1152/ajpendo.1980.238.5.E421 6990783

[phy214889-bib-0020] Dufer, M. , Haspel, D. , Krippeit‐Drews, P. , Aguilar‐Bryan, L. , Bryan, J. , & Drews, G. (2009). Activation of the Na^+^/K^+^‐ATPase by insulin and glucose as a putative negative feedback mechanism in pancreatic beta‐cells. Pflugers Archives: European Journal of Physiology, 457, 1351–1360. 10.1007/s00424-008-0592-4 18836740

[phy214889-bib-0021] Dutka, T. L. , & Lamb, G. D. (2007). Na^+^‐K^+^ pumps in the transverse tubular system of skeletal muscle fibers preferentially use ATP from glycolysis. American Journal of Physiology: Cell Physiology, 293, C967–C977. 17553934 10.1152/ajpcell.00132.2007

[phy214889-bib-0022] Erlij, D. , & Grinstein, S. (1976). The number of sodium ion pumping sites in skeletal muscle and its modification by insulin. Journal of Physiology, 259, 13–31. 10.1113/jphysiol.1976.sp011452 PMC1309012 182957

[phy214889-bib-0023] Ewart, H. S. , & Klip, A. (1995). Hormonal regulation of the Na(+)‐K(+)‐ATPase: Mechanisms underlying rapid and sustained changes in pump activity. American Journal of Physiology: Cell Physiology, 269, C295–C311. 10.1152/ajpcell.1995.269.2.C295 7653511

[phy214889-bib-0024] Ferrannini, E. , Taddei, S. , Santoro, D. , Natali, A. , Boni, C. , Del Chiaro, D. , & Buzzigoli, G. (1988). Independent stimulation of glucose metabolism and Na^+^‐K^+^ exchange by insulin in the human forearm. American Journal of Physiology: Endocrinology and Metabolism, 255, E953–E958. 10.1152/ajpendo.1988.255.6.E953 2849310

[phy214889-bib-0025] Fugmann, A. , Lind, L. , Andersson, P. E. , Millgard, J. , Hanni, A. , Berne, C. , & Lithell, H. (1998). The effect of euglucaemic hyperinsulinaemia on forearm blood flow and glucose uptake in the human forearm. Acta Diabetologica, 35, 203–206. 10.1007/s005920050132 9934819

[phy214889-bib-0026] Gagnon, K. B. , & Delpire, E. (2021). Sodium transporters in human health and disease. Frontiers in Physiology, 11, 588664. 10.3389/fphys.2020.588664 33716756 PMC7947867

[phy214889-bib-0027] Gavryck, W. A. , Moore, R. D. , & Thompson, R. C. (1975). Effect of insulin upon membrane‐bound (Na^+^ + K^+^)‐ATPase extracted from frog skeletal muscle. Journal of Physiology, 252, 43–58. 10.1113/jphysiol.1975.sp011133 PMC1348467 127836

[phy214889-bib-0028] Green, D. , Cheetham, C. , Mavaddat, L. , Watts, K. , Best, M. , Taylor, R. , & O'Driscoll, G. (2002). Effect of lower limb exercise on forearm vascular function: Contribution of nitric oxide. American Journal of Physiology: Heart and Circulatory Physiology, 283, H899–H907. 10.1152/ajpheart.00049.2002 12181117

[phy214889-bib-0029] Green, H. J. , Duhamel, T. A. , Foley, K. P. , Ouyang, J. , Smith, I. C. , & Stewart, R. D. (2007). Glucose supplements increase human muscle in vitro Na^+^‐K^+^‐ATPase activity during prolonged exercise. American Journal of Physiology: Regulatory, Integrative and Comparative Physiology, 293, R354–R362. 10.1152/ajpregu.00701.2006 17409263

[phy214889-bib-0030] Green, S. , Langberg, H. , Skovgaard, D. , Bulow, J. , & Kjar, M. (2000). Interstitial and arterial‐venous [K^+^] in human calf muscle during dynamic exercise: Effect of ischaemia and relation to muscle pain. Journal of Physiology, 529, 849–861. 10.1111/j.1469-7793.2000.00849.x PMC2270236 11118511

[phy214889-bib-0031] Hargreaves, M. , McKenna, M. J. , Jenkins, D. G. , Warmington, S. A. , Li, J. L. , Snow, R. J. , & Febbraio, M. A. (1998). Muscle metabolites and performance during high‐intensity, intermittent exercise. Journal of Applied Physiology, 84, 1687–1691. 10.1152/jappl.1998.84.5.1687 9572818

[phy214889-bib-0032] Harrison, M. H. (1985). Effects on thermal stress and exercise on blood volume in humans. Physiological Reviews, 65, 149–209. 10.1152/physrev.1985.65.1.149 3880897

[phy214889-bib-0033] Hundal, H. S. , Marette, A. , Mitsumoto, Y. , Ramlal, T. , Blostein, R. , & Klip, A. (1992). Insulin induces translocation of the alpha 2 and beta 1 subunits of the Na^+^/K^+^‐ATPase from intracellular compartments to the plasma membrane in mammalian skeletal muscle. The Journal of Biological Chemistry, 267, 5040–5043. 10.1016/S0021-9258(18)42725-1 1312081

[phy214889-bib-0034] James, J. H. , Fang, C. H. , Schrantz, S. J. , Hasselgren, P. O. , Paul, R. J. , & Fischer, J. E. (1996). Linkage of aerobic glycolysis to sodium‐potassium transport in rat skeletal muscle. Implications for increased muscle lactate production in sepsis. Journal of Clinical Investigation, 98, 2388–2397. 10.1172/JCI119052 PMC507691 8941658

[phy214889-bib-0035] Jensen, R. , Nielsen, J. , & Ørtenblad, N. (2020). Inhibition of glycogenolysis prolongs action potential repriming period and impairs muscle function in rat skeletal muscle. Journal of Physiology, 598, 789–803. 10.1113/JP278543 31823376

[phy214889-bib-0036] Juel, C. , Pilegaard, H. , Nielsen, J. J. , & Bangsbo, J. (2000). Interstitial K^+^ in human skeletal muscle during and after dynamic graded exercise determined by microdialysis. American Journal of Physiology: Regulatory, Integrative and Comparative Physiology, 278, R400–R406. 10.1152/ajpregu.2000.278.2.R400 10666141

[phy214889-bib-0037] Karelis, A. D. , Peronnet, F. , & Gardiner, P. F. (2002). Glucose infusion attenuates muscle fatigue in rat plantaris muscle during prolonged indirect stimulation in situ. Experimental Physiology, 87, 585–592. 10.1113/eph8702391 12481933

[phy214889-bib-0038] Karelis, A. D. , Peronnet, F. , & Gardiner, P. F. (2003). Insulin does not mediate the attenuation of fatigue associated with glucose infusion in rat plantaris muscle. Journal of Applied Physiology, 95, 330–335. 10.1152/japplphysiol.00040.2003 12639847

[phy214889-bib-0039] Kowalchuk, J. M. , Heigenhauser, G. J. , Lindinger, M. I. , Obminski, G. , Sutton, J. R. , & Jones, N. L. (1988). Role of lungs and inactive muscle in acid‐base control after maximal exercise. Journal of Applied Physiology, 65, 2090–2096. 10.1152/jappl.1988.65.5.2090 3145276

[phy214889-bib-0040] Lindinger, M. I. (1995). Potassium regulation during exercise and recovery in humans: Implications for skeletal and cardiac muscle. Journal of Molecular and Cellular Cardiology, 27, 1011–1022. 10.1016/0022-2828(95)90070-5 7563098

[phy214889-bib-0041] Lindinger, M. I. , & Cairns, S. P. (2021). Regulation of muscle potassium: Exercise performance, fatigue and health implications. European Journal of Applied Physiology, 121, 721–748. 10.1007/s00421-020-04546-8 33392745

[phy214889-bib-0042] Lindinger, M. I. , Heigenhauser, G. J. , McKelvie, R. S. , & Jones, N. L. (1990). Role of nonworking muscle on blood metabolites and ions with intense intermittent exercise. American Journal of Physiology: Regulatory, Integrative and Comparative Physiology, 258(6), R1486–R1494. 10.1152/ajpregu.1990.258.6.R1486 2360695

[phy214889-bib-0043] Lindinger, M. I. , Heigenhauser, G. J. , McKelvie, R. S. , & Jones, N. L. (1992). Blood ion regulation during repeated maximal exercise and recovery in humans. American Journal of Physiology: Regulatory, Integrative and Comparative Physiology, 262, R126–R136. 10.1152/ajpregu.1992.262.1.R126 1733331

[phy214889-bib-0044] Marette, A. , Krischer, J. , Lavoie, L. , Ackerley, C. , Carpentier, J. L. , & Klip, A. (1993). Insulin increases the Na^+^‐K^+^‐ATPase alpha 2‐subunit in the surface of rat skeletal muscle: Morphological evidence. American Journal of Physiology: Cell Physiology, 265, C1716–C1722. 10.1152/ajpcell.1993.265.6.C1716 8279532

[phy214889-bib-0045] McConell, G. , Fabris, S. , Proietto, J. , & Hargreaves, M. (1994). Effect of carbohydrate ingestion on glucose kinetics during exercise. Journal of Applied Physiology, 77, 1537–1541. 10.1152/jappl.1994.77.3.1537 7836162

[phy214889-bib-0046] McDonough, A. A. , & Youn, J. H. (2017). Potassium homeostasis: The knowns, the unknowns, and the health benefits. Physiology, 32, 100–111. 10.1152/physiol.00022.2016 28202621 PMC5337831

[phy214889-bib-0047] McGill, D. L. , & Guidotti, G. (1991). Insulin stimulates both the alpha 1 and the alpha 2 isoforms of the rat adipocyte (Na^+^,K^+^) ATPase. Two mechanisms of stimulation. The Journal of Biological Chemistry, 266, 15824–15831. 10.1016/S0021-9258(18)98482-6 1651922

[phy214889-bib-0048] McKenna, M. J. (1992). The roles of ionic processes in muscular fatigue during intense exercise. Sports Medicine, 13, 134–145. 1373245 10.2165/00007256-199213020-00009

[phy214889-bib-0049] McKenna, M. J. , Bangsbo, J. , & Renaud, J. M. (2008). Muscle K^+^, Na^+^, and Cl disturbances and Na^+^‐K^+^ pump inactivation: Implications for fatigue. Journal of Applied Physiology, 104, 288–295. 17962569 10.1152/japplphysiol.01037.2007

[phy214889-bib-0050] McKenna, M. J. , Gissel, H. , & Clausen, T. (2003). Effects of electrical stimulation and insulin on Na^+^,K^+^‐ATPase ([^3^H]‐ouabain binding) in rat skeletal muscle. Journal of Physiology, 547, 567–580. 10.1113/jphysiol.2003.034512 PMC2342648 12562912

[phy214889-bib-0051] McKenna, M. J. , Heigenhauser, G. J. , McKelvie, R. S. , MacDougall, J. D. , & Jones, N. L. (1997). Sprint training enhances ionic regulation during intense exercise in men. Journal of Physiology, 501(Pt 3), 687–702. 10.1111/j.1469-7793.1997.687bm.x PMC1159469 9218228

[phy214889-bib-0052] Medbø, J. I. , Hisdal, J. , & Stranden, E. (2009). Blood flow in the brachial artery increases after intense cycling exercise. Scandinavian Journal of Clinical and Laboratory Investigation, 69, 752–763. 10.3109/00365510903128558 19929718

[phy214889-bib-0053] Medved, I. , Brown, M. J. , Bjorksten, A. R. , Leppik, J. A. , Sostaric, S. , & McKenna, M. J. (2003). N‐acetylcysteine infusion alters blood redox status but not time to fatigue during intense exercise in humans. Journal of Applied Physiology, 94, 1572–1582. 12496140 10.1152/japplphysiol.00884.2002

[phy214889-bib-0054] Moore, R. D. (1973). Effect of insulin upon the sodium pump in frog skeletal muscle. Journal of Physiology, 232, 23–45. 10.1113/jphysiol.1973.sp010255 PMC1350490 4542575

[phy214889-bib-0055] Murray, R. , Eddy, D. E. , Murray, T. W. , Seifert, J. G. , Paul, G. L. , & Halaby, G. A. (1987). The effect of fluid and carbohydrate feedings during intermittent cycling exercise. Medicine and Science in Sports and Exercise, 19, 597–604. 10.1249/00005768-198712000-00010 3431377

[phy214889-bib-0056] Muto, S. , Sebata, K. , Watanabe, H. , Shoji, F. , Yamamoto, Y. , Ohashi, M. , Yamada, T. , Matsumoto, H. , Mukouyama, T. , Yonekura, T. , Namiki, S. , & Kusano, E. (2005). Effect of oral glucose administration on serum potassium concentration in hemodialysis patients. American Journal of Kidney Disease, 46, 697–705. 10.1053/j.ajkd.2005.06.013 16183425

[phy214889-bib-0057] Natali, A. , Quinones Galvan, A. , Santoro, D. , Pecori, N. , Taddei, S. , Salvetti, A. , & Ferrannini, E. (1993). Relationship between insulin release, antinatriuresis and hypokalaemia after glucose ingestion in normal and hypertensive man. Clinical Science, 85, 327–335. 10.1042/cs0850327 8403806

[phy214889-bib-0058] Nordsborg, N. , Mohr, M. , Pedersen, L. D. , Nielsen, J. J. , Langberg, H. , & Bangsbo, J. (2003). Muscle interstitial potassium kinetics during intense exhaustive exercise: Effect of previous arm exercise. American Journal of Physiology: Regulatory, Integrative and Comparative Physiology, 285, R143–R148. 10.1152/ajpregu.00029.2003 12663256

[phy214889-bib-0059] Novel‐Chate, V. , Rey, V. , Chiolero, R. , Schneiter, P. , Leverve, X. , Jequier, E. , & Tappy, L. (2001). Role of Na^+^‐K^+^‐ATPase in insulin‐induced lactate release by skeletal muscle. American Journal of Physiology: Endocrinology and Metabolism, 280, E296–E300. 11158933 10.1152/ajpendo.2001.280.2.E296

[phy214889-bib-0060] Okamoto, K. , Wang, W. , Rounds, J. , Chambers, E. A. , & Jacobs, D. O. (2001). ATP from glycolysis is required for normal sodium homeostasis in resting fast‐twitch rodent skeletal muscle. American Journal of Physiology: Endocrinology and Metabolism, 281, E479–E488. 11500303 10.1152/ajpendo.2001.281.3.E479

[phy214889-bib-0061] Poortmans, J. R. , Delescaille‐Vanden Bossche, J. , & Leclercq, R. (1978). Lactate uptake by inactive forearm during progressive leg exercise. Journal of Applied Physiology, 45, 835–839. 10.1152/jappl.1978.45.6.835 730585

[phy214889-bib-0062] Sampson, S. R. , Brodie, C. , & Alboim, S. V. (1994). Role of protein kinase C in insulin activation of the Na‐K pump in cultured skeletal muscle. American Journal of Physiology: Cell Physiology, 266, C751–C758. 10.1152/ajpcell.1994.266.3.C751 8166238

[phy214889-bib-0063] Sejersted, O. M. , & Sjøgaard, G. (2000). Dynamics and consequences of potassium shifts in skeletal muscle and heart during exercise. Physiological Reviews, 80, 1411–1481. 10.1152/physrev.2000.80.4.1411 11015618

[phy214889-bib-0064] Sostaric, S. M. , Skinner, S. L. , Brown, M. J. , Sangkabutra, T. , Medved, I. , Medley, T. , Selig, S. E. , Fairweather, I. , Rutar, D. , & McKenna, M. J. (2006). Alkalosis increases muscle K^+^ release, but lowers plasma [K^+^] and delays fatigue during dynamic forearm exercise. Journal of Physiology, 570, 185–205. 10.1113/jphysiol.2005.094615 PMC1464289 16239279

[phy214889-bib-0065] Stainsby, W. N. , Brechue, W. F. , & O'Drobinak, D. M. (1991). Regulation of muscle lactate production. Medicine and Science in Sports and Exercise, 23, 907–911. 10.1249/00005768-199108000-00004 1956263

[phy214889-bib-0066] Tanaka, H. , Shimizu, S. , Ohmori, F. , Muraoka, Y. , Kumagai, M. , Yoshizawa, M. , & Kagaya, A. (2006). Increases in blood flow and shear stress to nonworking limbs during incremental exercise. Medicine and Science in Sports and Exercise, 38, 81–85. 10.1249/01.mss.0000191166.81789.de 16394957

[phy214889-bib-0067] Tarnopolsky, M. A. , Dyson, K. , Atkinson, S. A. , MacDougall, D. , & Cupido, C. (1996). Mixed carbohydrate supplementation increases carbohydrate oxidation and endurance exercise performance and attenuates potassium accumulation. International Journal of Sport Nutrition, 6, 323–336. 10.1123/ijsn.6.4.323 8953335

[phy214889-bib-0068] Zierler, K. L. , & Rabinowitz, D. (1964). Effect of very small concentrations of insulin on forearm metabolism. Persistence of its action on potassium and free fatty acids without its effect on glucose. Journal of Clinical Investigation, 43, 950–962. 10.1172/JCI104981 PMC289574 14169524

